# Screening and identification of emodin as an EBV DNase inhibitor to prevent its biological functions

**DOI:** 10.1186/s12985-023-02107-x

**Published:** 2023-07-13

**Authors:** Chung-Chun Wu, Mei-Shu Chen, Ting-Ying Lee, Yu-Jhen Cheng, Hsiao-Hui Tsou, Tze-Sing Huang, Der-Yang Cho, Jen-Yang Chen

**Affiliations:** 1 Translational Cell Therapy Center, Department of Medical Research, China Medical University Hospital, No. 2, Yude Rd., North Dist, Taichung City, 40447 Taiwan; 2 National Institute of Cancer Research, National Health Research Institutes, No.35, Keyan Road, Zhunan Town, Miaoli County Taiwan; 3 National Institute of Infectious Diseases and Vaccinology, National Health Research Institutes, Zhunan, Taiwan; 4 Institute of Population Health Sciences, National Health Research Institutes, Zhunan, Taiwan

**Keywords:** EBV DNase, BGLF5, Alkaline nuclease, PicoGreen, Fluorescence

## Abstract

**Background:**

The Epstein-Barr virus (EBV) is a prevalent oncovirus associated with a variety of human illnesses. BGLF5, an EBV DNase with alkaline nuclease (AN) activity, plays important roles in the viral life cycle and progression of human malignancies and has been suggested as a possible diagnostic marker and target for cancer therapy. Methods used conventionally for the detection of AN activity, radioactivity-based nuclease activity assay and DNA digestion detection by gel electrophoresis, are not suitable for screening AN inhibitors; the former approach is unsafe, and the latter is complicated. In the present study, a fluorescence-based nuclease activity assay was used to screen several natural compounds and identify an EBV DNase inhibitor.

**Results:**

Fluorescence-based nuclease activity assays, in which the DNA substrate is labelled with PicoGreen dye, are cheaper, safer, and easier to perform. Herein, the results of the fluorescence-based nuclease activity assay were consistent with the results of the two conventional methods. In addition, the PicoGreen-labelling method was applied for the biochemical characterisation of viral nucleases. Using this approach, we explored EBV DNase inhibitors. After several rounds of screening, emodin, an anthraquinone derivative, was found to possess significant anti-EBV DNase activity. We verified the efficacy of emodin using the conventional DNA-cleavage assay. Furthermore, using comet assay and micronucleus formation detection, we confirmed that emodin can inhibit DNase-induced DNA damage and genomic instability. Additionally, emodin treatment inhibited EBV production.

**Conclusions:**

Using a PicoGreen-mediated nuclease activity assay, we successfully demonstrated that emodin has the potential to inhibit EBV DNase nuclease activity. Emodin also inhibits EBV DNase-related biological functions, suggesting that it is a potential inhibitor of EBV DNase.

**Supplementary Information:**

The online version contains supplementary material available at 10.1186/s12985-023-02107-x.

## Introduction

Alkaline nucleases (ANs), which are named for their ability to degrade DNA under alkaline conditions, are found ubiquitously in human herpesviruses, including herpes simplex virus 1 (HSV-1) [30], varicella-zoster virus [16], cytomegalovirus (CMV) [47], Epstein-Barr virus (EBV) [15], and Kaposi’s sarcoma-associated herpesvirus (KSHV) [21]. ANs have several conserved domains that catalyse DNA cleavage and binding [38]. Metal ions are required for DNA cleavage and for different catalytic properties and cleavage targets [5, 38]. Among ANs, EBV DNase (BGLF5) is one of the most well studied ANs and has been found to have several unique features in viral biology. During the viral life cycle, EBV DNase is important for the generation and processing of linear viral genomes [20]. With respect to carcinogenesis, serological evidence indicated that patients with nasopharyngeal carcinoma (NPC) have higher titres of antibodies against EBV DNase than healthy controls [14] and antibody titres were raised prior to the appearance of clinical symptoms of NPC [11]. Histopathological surveys have shown that significant amounts of EBV DNase protein and nuclease activity are detected in fresh biopsies and transplanted tumour lines [43]. In cancer biology studies, EBV DNase has been suggested to be a mutagen with the ability to induce cellular genomic instability, cleaving DNAs, and indirectly repressing DNA repair [56]. In addition, disrupted nuclease activity inhibits its ability to induce genomic instability [56]. Furthermore, EBV DNase degrades mRNA to block the synthesis of human leukocyte antigen class I and II, which results in the immune evasion of EBV [42]. Based on these observations, EBV DNase is a possible target for anti-carcinogenic and anti-virus purposes and it is worth exploring nuclease inhibitors against EBV DNase activity to develop alternative anti-viral or -cancer therapies. In addition to EBV DNase, the inhibition of other herpesviral nucleases, such as UL12 of HSV-1, UL98 of CMV, and Sox of KSHV, have been identified as potential targets for anti-viral therapy. Therefore, identification of AN inhibitors is an attractive strategy for drug discovery.

For both safety and convenience, natural compounds are a useful source to identify AN inhibitors. The anthraquinone emodin was reported to inhibit the nuclease activity of HSV-1 AN and decreased HSV-1 virus yields [32]. Similarly, another anthraquinone Atanyl blue PRL also inhibits the nuclease activities of CMV UL98 [1] and HSV-1 UL12 [22] and blocking viral production. Recently, metal-directed hydroxytropolones have been found to repress HSV-1 AN activity [22] and suppress HSV infection [51]. These studies revealed that natural compounds represent a promising source for identifying AN inhibitors.

Prior to screening potential AN inhibitors, the method employed for the detection of AN activity needs to be considered. Several methods have been proposed for detecting AN activity, including a radioisotope-based nuclease activity assay [14, 15, 29]. In this assay, the DNA substrate is radiolabelled by growing thymine-dependent *Escherichia coli* in a ^14^C-thymine-containing medium or directly labelled with ^32^P using Klenow polymerase. DNase digestion releases radioactive nucleotides from the substrate DNA and can be detected based on their acid solubility in the reaction mixture. Radioactivity in the supernatant reflects the DNase activity. Radioisotope-based methods allow easy quantification; however, they are unsafe and inconvenient due to isotopic materials. Another common method for the detection of DNA degradation, including DNA cleavage by nucleases, is agarose gel electrophoresis [31, 42]; however, it is difficult to quantify DNase activity with this approach and the protocol needs many steps. In addition to these methods, fluorescence-based assays have also been developed [6, 33]. This approach quantifies the amount of double-stranded (ds) DNA using a fluorescent reagent and has good sensitivity and specificity for detecting double-stranded (ds) DNA in solution, along with being safe and convenient [48].

In the present study, a fluorescence densitometric assay was developed and established using PicoGreen dye to detect nuclease activity. Furthermore, using this assay, we identified emodin as a potential AN inhibitor that inhibits EBV DNase activity. Our findings suggest that emodin may be an alternative choice for anti-viral therapy and is worthy of further study.

## Materials and methods

### Cell culture

TW01 is an Epstein-Barr virus-negative NPC cell line [34]. NA is an EBV-positive NPC cell line that is derived from the EBV infection into TW01 cells [9]. NPC cell lines were cultured in Dulbecco's modified eagle medium supplemented with 10% foetal bovine albumin with (NA) or without (TW01) 400 μg/mL G418 (Sigma-Aldrich, St. Louis, MO, USA).

### Reagents and antibodies

Emodin, 12-O-tetradecanoyl-phorbol-1,3-acetate (TPA), and sodium butyrate (SB) were purchased from Sigma-Aldrich. PicoGreen was purchased from Thermo Fisher Scientific (Waltham, MA, USA). The EBV DNase antibody used in this study was anti-DNase 311H [52].

### Plasmid construction and mutagenesis

To express recombinant EBV DNase and mutants in *E. coli,* the plasmid encoding the wild-type EBV DNase BGLF5 sequence (P3HR1 strain, 470 amino acids) was subcloned into the vector pET-15b [35]. Mutants were generated using site-directed mutagenesis strategies established previously [37]. Mutants with the E225A single mutation (mut E225A) or E225A/K227A double mutations were generated by site-directed mutagenesis and subcloned into pET-15b [37].

To express EBV DNase in NPC TW01 cells, BGLF5 sequences were subcloned into the pEGFP-CPO-IRES-puro vector, which was derived from pEGFP-C1 by CPO site ligation [56].

### Expression and purification of his-tagged recombinant EBV DNase and mutants

Expression and purification of EBV DNase were performed as described previously [38]. Briefly, pET-15b-driven EBV DNase or mutants were transformed into *E. coli* BL21(DE3) pLysS. At OD600 0.4 ~ 0.6, IPTG (1 mM) was added to the culture for 2 h. Cells were harvested by centrifugation, and cell pellets were resuspended in binding buffer (5 mM imidazole, 500 mM NaCl, 20 mM Tris/HCl, pH 8.0, 0.1% NP-40 and 10% glycerol). After sonication and centrifugation at 39 000 g for 20 min, the supernatant was used as the starting material for DNase purification, and subjected to a His-Bind column (Novagen, Pretoria, South Africa). The column was washed sequentially with two times of wash buffer 1 (20 mM Tris/HCl, pH 8.0, 500 mM NaCl, 10% glycerol, 20 mM imidazole), two times of wash buffer 2 (20 mM Tris/HCl, pH 8.0, 10% glycerol, 50 mM imidazole), and five times of elution buffer with increasing imidazole (20 mM Tris–HCl, pH 8.0, 10% glycerol, 100 ~ 500 mM imidazole). The purified proteins were analysed by sodium dodecyl sulphate – polyacrylamide gel electrophoresis (SDS–PAGE) (Additional file 1: Figure S1), quantified with a Bradford assay kit (Bio-Rad, Hercules, CA, USA), and then stored at − 70 °Ϲ until future use.

### DNA cleavage assay

For DNA cleavage assay, 500 ng of purified EBV DNase mixed with 0.5 mg of the plasmid DNA pEGFP-C1 (Invitrogen) in reaction buffer (50 mM Tris/HCl, pH 8.0, 4 mM b-mercaptoethanol and 4 mM MgCl_2_) incubated at 37 °C for 1 h. The mixtures were analysed by agarose gel electrophoresis.

### Nuclease activity assay using isotope substrate

In the isotope-based method, AN activity was measured by assessing the release of soluble nucleotides from isotope-labelled DNA (Additional file 1: Figure S2a) [29]. To perform the radioactivity-based assay, C^14^-labelled calf thymus DNA was used as the DNA substrate. Purified recombinant proteins of wild-type and mutant EBV DNase (1 μg) were diluted with dilution buffer (50 mM Tris–HCl, pH 8.0, 10 mM MgCl_2_, 10 mM 2-mercaptoethanol, 0.05% bovine serum albumin), and then were incubated with 1 μg C^14^-labelled DNA for 1 h at 37 °Ϲ. After adding the stop solution (50% TCA and sheared calf thymus DNA), the sample was spun down and the radioactivity of supernatant was measured using a liquid scintillation counter (LS-6000; Beckman, Fullerton, CA, USA). Enzyme activity was defined as the percentage of DNA hydrolysed, which was calculated as the count per minute (cpm) of each sample normalised to the total amount of isotope usage.

### Nuclease activity assay using PicoGreen reagent

For the PicoGreen-labelled method, AN activity was assessed using PicoGreen-incorporated double-stranded DNA, as described in previous studies (Additional file 1: Figure S2b). Briefly, purified recombinant proteins (1 μg) were diluted with dilution buffer (50 mM Tris–HCl, pH 8.0, 10 mM MgCl2, 10 mM 2-mercaptoethanol, 0.05% BSA). The diluted solutions were mixed with 90 ng salmon sperm DNA, and adjusted with various concentrations of metal ions, pH, or other conditions for 1 h at 37 °Ϲ, respectively. To determine the influence of pH, reaction buffers of pH 7–9 were prepared with 50 mM Tris–HCl, pH 9.5–10.5 with 25 mM borate buffer, and pH 11–11.5 with sodium bicarbonate-NaOH. After then, the samples were mixed with 100 μl PicoGreen dye (Molecular Probe, Waltham, MA, USA; 200-fold diluted in 10 mM Tris–HCl, pH 8.0, and 10 mM EDTA) to label DNA, then incubated the mixture of samples and PicoGreen dye for 2 to 5 min, and finally, fluorescence (Excitation: 480 nm; Emission: 520 nm) was detected using a microplate reader (Infinite M 200, Tecan, Männedorf, Switzerland). Enzyme activity was defined as the percentage of DNA hydrolysed, which was calculated as follows: the fluorescent read of total labelled DNA was subtracted from that of the fluorescent read of each sample, and the result was normalised to the fluorescent read of total labelled DNA.

For screening natural compounds against AN activity, purified recombinant proteins (1 μg) were diluted with a dilution buffer. The diluted solutions were mixed with 90 ng salmon sperm DNA and indicated amount of natural compounds for 1 h at 37 °Ϲ. After then, the samples were mixed with 100 μl PicoGreen dye as the same as described above. The fluorescence (Excitation: 480 nm; Emission: 520 nm) was detected using a microplate reader (Infinite M 200, Tecan, Männedorf, Switzerland). The IC50 value of each compound was calculated by the Microsoft Excel or SigmaPlot using the linear regression.

### Transfection

For TW01 transfection, plasmid was transfected using Lipofectamine 2000 (Invitrogen), according to the manufacturer’s instructions. Briefly, TW01 cells were plated for 24 h. Before transfection, the plasmid DNA was mixed with Lipofectamine 2000 in Opti-MEM medium (Invitrogen) and incubated for 20 min, then subjected to the culture well containing TW01 cells.

### Micronucleus (MN) formation assay

MN formation was detected as described previously [25, 36, 56]. Briefly, TW01 cell lines transfected by lipofectamine 2000 (Invitrogen) with EBV DNase-expressing plasmids or parental vectors pEGFP-CPO-IRES-puro were plated on cover slides, with or without emodin treatment. After 24 h of incubation, the cells were washed with phosphate-buffered saline (PBS) (pH 7.4) and fixed with cold methanol for 15 min. After washing with PBS, the cells were stained with Hoechst dye (0.2 μg/mL, Sigma-Aldrich, St. Louis, MO, USA) for 15 min. The MN was counted under a fluorescence microscope. The percentage of MN formation was defined as the number of MN divided by the total number of counted cells; at least 1000 cells were counted in each group. The IC50 value of each sample was calculated by the Microsoft Excel or SigmaPlot using the non-linear regression.

### Western blotting

Western blotting was performed as described previously [38]. Briefly, purified EBV DNase and its mutants were subjected to SDS–PAGE. The proteins were transferred to Hybond-C membranes (Amersham Biosciences, Amersham, United Kingdom). The membranes were blocked with 4% milk for 1 h and incubated with 1:1 diluted EBV DNase antibodies [52] overnight at 4 °Ϲ. After washing with the wash buffer (10 mM Tris–HCl, pH 8.0, 0.9% NaCl), the membranes were incubated with a secondary antibody (horseradish peroxidase-labeled goat anti-mouse IgG, Amersham, diluted 1:2500) and developed using a developing substrate (Amersham Biosciences Ltd.). Luminescence was detected by exposure to an X-ray film.

### Single-cell gel electrophoresis (comet assay)

Comet assay was used to detect DNA damage [56], according to the manufacturer’s instructions (Trevigen, Minneapolis, MN, USA). Briefly, the cells were transfected with the vector or DNase-expressing plasmids for 3 h. Next, emodin was added, and the cells were incubated for 24 h. The treated cells (1 × 10^5^/ml) were harvested and resuspended in a 1:10 (v/v) mixture of PBS and molten low-melting agarose (Trevigen, Minneapolis, MN, USA) at 37 °Ϲ for a total of 100 μl. The mixture (75 μl) was immediately pipetted onto CometSlideTM (Trevigen, Minneapolis, MN, USA). After cooling, the slides were treated with lysis buffer and subsequently with an alkaline solution (300 mM NaOH and 1 mM EDTA) to denature cellular DNA. The samples were subjected to electrophoresis and stained with SYBR Green to observe comet tails using a fluorescence microscope. One hundred comets were scored randomly. Each comet was assigned a visual score of 0–4 based on the tail length. The length and intensity of comet tails are positively correlated to the visual scores. The standards of scoring are: class 0 is a score of 0, class 1 is a score of 1, and so on. The total visual score was calculated to determine the level of DNA damage [3, 40]. The IC50 value of each sample was calculated by the Microsoft Excel or SigmaPlot using the non-linear regression.

### Determination of the copy number of the EBV genome

To evaluate the production of EBV, the copy number of the EBV genome in the released particles was determined as previously described [13, 55]. Briefly, NA cells were pre-treated with emodin for 1 h prior to TPA (40 ng/mL) and SB (3 mM) treatment for 48 h. Supernatants were filtered through a 0.45 μM filter and treated with DNase I and DNase I buffer (10 mM Tris–HCl, 2.5 mM MgCl2, 0.5 mM CaCl2, pH 7.6) at 37 °Ϲ for 60 min. Subsequently, EDTA (2 mM, pH 8.0) was added to terminate the reaction, and each sample was treated with proteinase K (0.1 mg/mL, sample: proteinase K = 1:1 [vol/vol]) at 50 °Ϲ for 60 min. Proteinase K was then inactivated at 75 °Ϲ for 20 min. Subsequently, real-time PCR analysis [13] was performed to detect the BALF5 fragment using the following primers: sense, 5′-CGGAGTTGTTATCAAAGAGGC-3′; antisense, 5′-CGAGAAAGACGGAGATGGC-3′. The qPCR cycling conditions were as follows: denaturation for 5 s at 95 °Ϲ, annealing for 20 s at 60 °Ϲ, and extension for 2 s at 72 °Ϲ for 45 cycles in the LightCycler 480 (Roche Applied Science, Indianapolis, IN, USA). NA cells activated by TPA + SB without emodin were used as a positive control, whereas cells that were not treated with any compounds were used as mock controls. The relative fold change in viral production was defined as the qPCR value of each sample normalised to the individual cell numbers divided by that of the mock control. The EC50 value of each sample was calculated by the Microsoft Excel or SigmaPlot using the non-linear regression.

### The prediction of the docking model between EBV DNase and emodin

The protein structure of EBV DNase BGLF5 was obtained from the NCBI server (https://www.ncbi.nlm.nih.gov/Structure/pdb/2W45) [7]. The software Swissdock [23, 24] and UCSF Chimera [41] were used for predicting a possible docking model between EBV DNase and emodin. The input files were in the PDB formats. The PDB format of emodin was obtained from the PubChem server (https://pubchem.ncbi.nlm.nih.gov/compound/Emodin).

## Results

### The development of a fluorescence-based method to detect AN activity of EBV DNase

According to previous studies, EBV DNase possesses a catalytic site for the degradation of DNA substrates, which is conserved in other herpesviral ANs and is homologous to that of type II restriction endonucleases [31, 38]. This core region is composed of D203…E225XK227 (D…EXK), among which, D203 is important for metal ion coordination, E225 for coordinating metal ions DNA binding, and K227 for DNA binding [38].

**Fig. 1:**
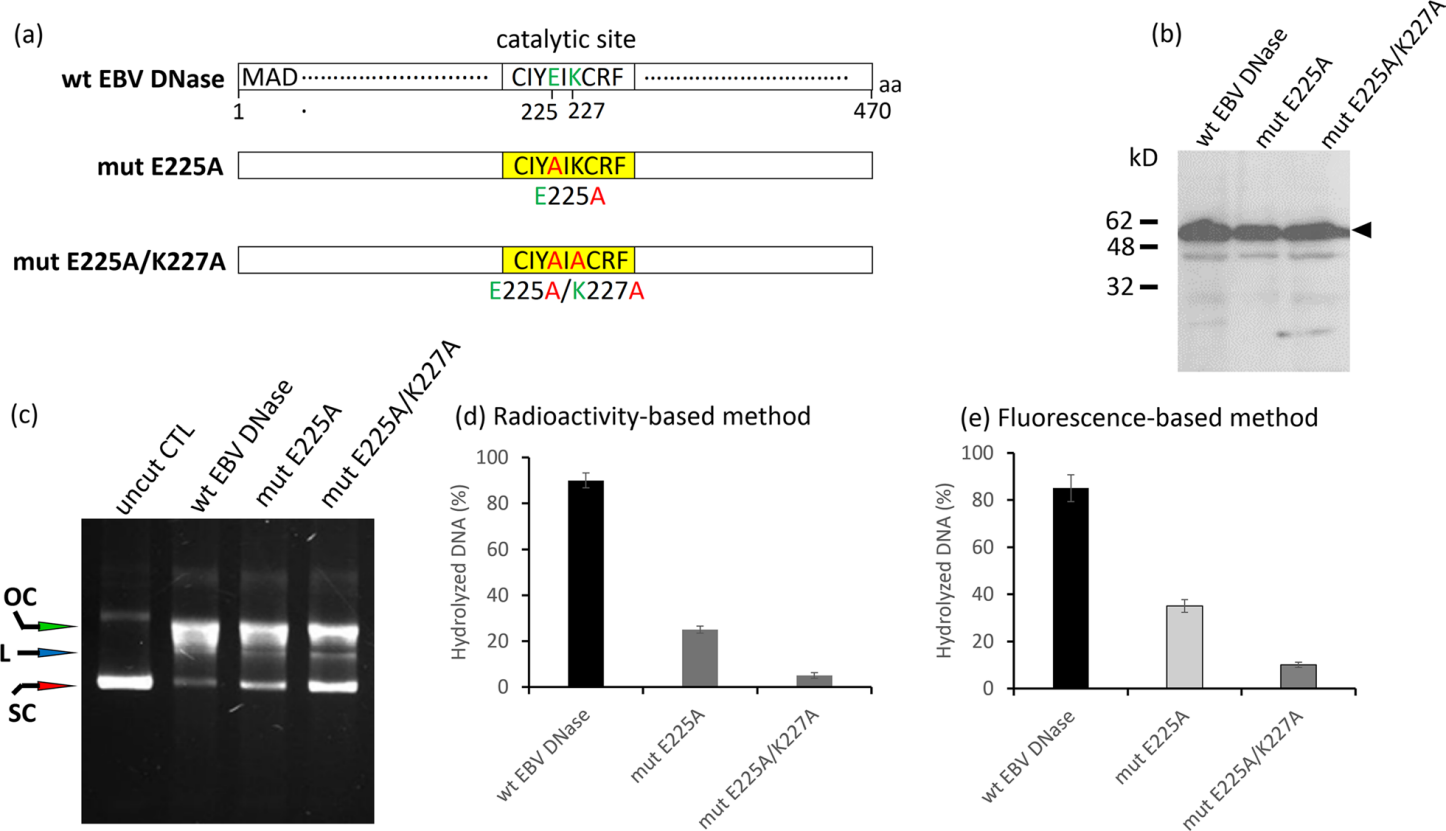
Detection of enzymatic activities of EBV DNase by different approaches. **a** Three purified recombinant proteins, wild-type EBV DNase and two mutants (mut E225A and mut E225A/K227A) driven by pET-15b vectors and expressed in *E. coli* BL21 (DE3) pLysS, were subjected to detection of AN activity using three detection methods: DNA cleavage analysis, isotope-based assay and fluorescence-based method. **b** Wild-type (wt), E225A (mut E225A) and E225A/K227A (mut E225A/K227A) proteins were purified through a Ni-chelating column and assessed by western blot analysis. The arrow indicates the position of the EBV DNase proteins. **c** Conventional DNA cleavage assay was performed to detect the AN activity of three purified proteins. Proteins were incubated with circular DNA plasmid pEGFP-C1 for 1 h at 37 °Ϲ. Samples were analysed by 0.7% agarose electrophoresis. OC, L and SC represent the respective positions of the open circular, linear and supercoiled forms of the pEGFP-C1 plasmid. **d** Radioactivity-based approach was used for the detection of AN activity of EBV DNase and mutants digesting for 1 h at 37 °Ϲ. **e** Fluorescence-based approach was used for the detection of AN activity of EBV DNase and mutants digesting for 1 h at 37 °Ϲ. Data are presented as the means and standard deviations of three independent experiments

To establish a fluorescence-based method to detect AN activity, the purified recombinant proteins of the wild-type EBV DNase (wt EBV DNase) and its two mutant derivatives (mut E225A and mut E225A/K227A) derived from *E. coli* BL21(DE3) strain pLysS were used to detect nuclease activity (Fig. 1a) [37]. The protein status in the three clones was examined using western blot analysis (Fig. 1b). Nuclease activity was examined using three approaches, including DNA cleavage analysis, isotope-based assay and fluorescence-based methods. As shown in Fig. 1c, the decrease in band intensity revealed that wt EBV DNase had strong activity, whereas the mut E225A retained moderate activity, and mut E225A/K227A retained weak activity which was consistent with our previous results [37]. The results of the isotope-based assay were similar to those of the DNA-cutting analysis (Fig. 1d). Furthermore, the results of the fluorescence-based method were consistent with those of conventional approaches (Fig. 1e). These results suggest that the fluorescence-based assay was successfully developed and is ready for further examination.

### The effect of pH, temperature, divalent cations, and ion strength on EBV DNase AN activity

**Fig. 2:**
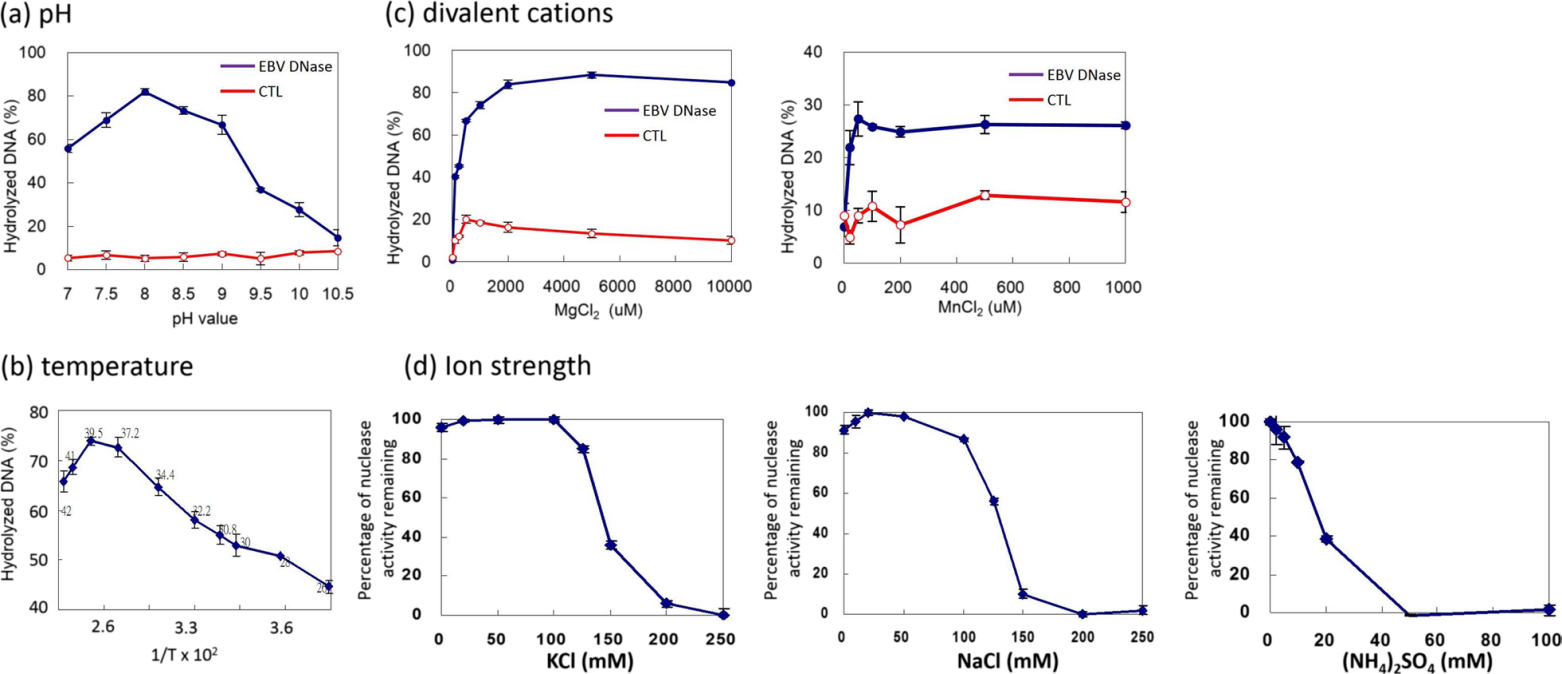
The effects of pH, temperature, divalent cations, and ion strength on EBV DNase activity. The biochemical characteristics of the purified EBV DNase were assessed using the fluorescence-based method. **a** To determine the optimal pH, proteins were diluted in different pH conditions and were incubated with salmon sperm DNA for 1 h at 37 °Ϲ. PicoGreen dye was then added and fluorescence was detected using a microplate reader. Solid squares (EBV DNase) represent EBV DNase groups and open circles (CTL) represent mock controls without enzymes, respectively. **b** To determine the optimum temperature, the protocol was the same as described above, except for incubation at different temperatures. **c** To determine the optimal concentration of divalent cations for EBV DNase, nuclease activity was assessed following incubation of DNase protein with salmon sperm DNA with various concentrations of magnesium (left panel) and manganese (right panel). Solid squares (EBV DNase) represent EBV DNase groups and open circles (CTL) represent mock controls without enzymes, respectively. **d** To identify the inhibition of nuclease activity of EBV DNase at different ionic strengths, DNase protein was incubated with salmon sperm DNA at various concentrations of KCl (left panel), NaCl (middle panel), and (NH_4_)_2_SO_4_ (right panel). PicoGreen dye was then added, and fluorescence was detected (excitation: 480 nm, emission: 520 nm). Data are presented as the means and standard deviations of three independent experiments

The catalytic environment is important for nuclease cleavage. Therefore, we determined whether the PicoGreen-labelled method could be used to detect the effect of environmental conditions on EBV DNase activity. The following environmental factors are known to affect the nuclease activity of EBV DNase: alkaline condition (pH 8 ~ 9), 37 °Ϲ and the presence of Mg^2+^ ion [12, 30, 43]. As shown in Fig. 2a, EBV DNase exhibited maximum activity at pH 8.0, whereas the control reactions showed no detectable activity. This result fits the existing knowledge about herpesviral nucleases and is consistent with the terminology “alkaline nucleases” [12, 43].

Next, to determine the optimal temperature, reactions were carried out at the optimum pH (pH 8.0) at various temperatures. As shown in Fig. 2b, EBV had the best performance at 37.2–39.5 °Ϲ, which is close to the preferred reaction temperature of EBV DNase, 37 °Ϲ, reported in previous studies [12, 43]. Divalent metal ions, especially magnesium (Mg^2+^) and manganese (Mn^2+^) are essential for the catalysis of DNA degradation [5], including those herpesviral nucleases [30, 47, 50]. To determine the optimal concentration of divalent cations for EBV DNase using the developed fluorescence-based approach, various concentrations of Mg^2+^ and Mn^2+^ were used. The CTL group is in the same reaction condition without EBV DNase. The results showed that EBV DNase lost its activity in the absence of either magnesium or manganese, suggesting that divalent cations are required for enzymatic activity (Fig. 2c, left and right panels). In contrast, EBV nuclease activity increased with increasing magnesium concentration and maximum activity was seen at 5 mM Mg^2+^ (Fig. 2c, left panel). For manganese, EBV DNase had a preferential Mn^2+^ concentration of 50 μM (Fig. 2c, right panel). Interestingly, considering the optimal activity for DNA hydrolysis, EBV DNase hydrolyzed 88% of substrate DNA at the highest Mg^2+^ concentration tests compared to 28% at the highest Mn^2+^ concentration tests (Fig. 2c, left and right panels), suggesting EBV DNase had lower efficiency of DNA hydrolysis with Mn^2+^ compare to that with Mg^2+^.

Furthermore, ionic strength is important for herpesviral nuclease activity. KCl, NaCl and (NH_4_)_2_SO_4_ have been reported to exhibit an inhibitory effect on herpesviral nucleases [4, 29, 50]. To determine whether these phenomena could be detected using the fluorescence-based assay, salts were added to the reaction mixtures at various concentrations. As expected, EBV DNase nuclease activity was inhibited with increasing concentrations of KCl, with an IC_50_ value of 140 mM (Fig. 2d, left panel). Consistently, EBV DNase was resistant to high concentrations of NaCl (IC_50_ = 125 mM) (Fig. 2d, middle panel). In contrast, with (NH_4_)_2_SO_4_, EBV DNase was inhibited significantly, with an IC_50_ value of 15 mM (Fig. 2d, right panel). These results indicate that the fluorescence-based assay can determine the sensitivity of EBV DNase to high ionic strengths.

Taken together, these results suggest that the fluorescence-based nuclease activity assay can be used to detect the preferred environmental conditions for EBV DNase activity.

### The inhibitory effects of natural compounds on nuclease activity of EBV DNase

**Fig. 3:**
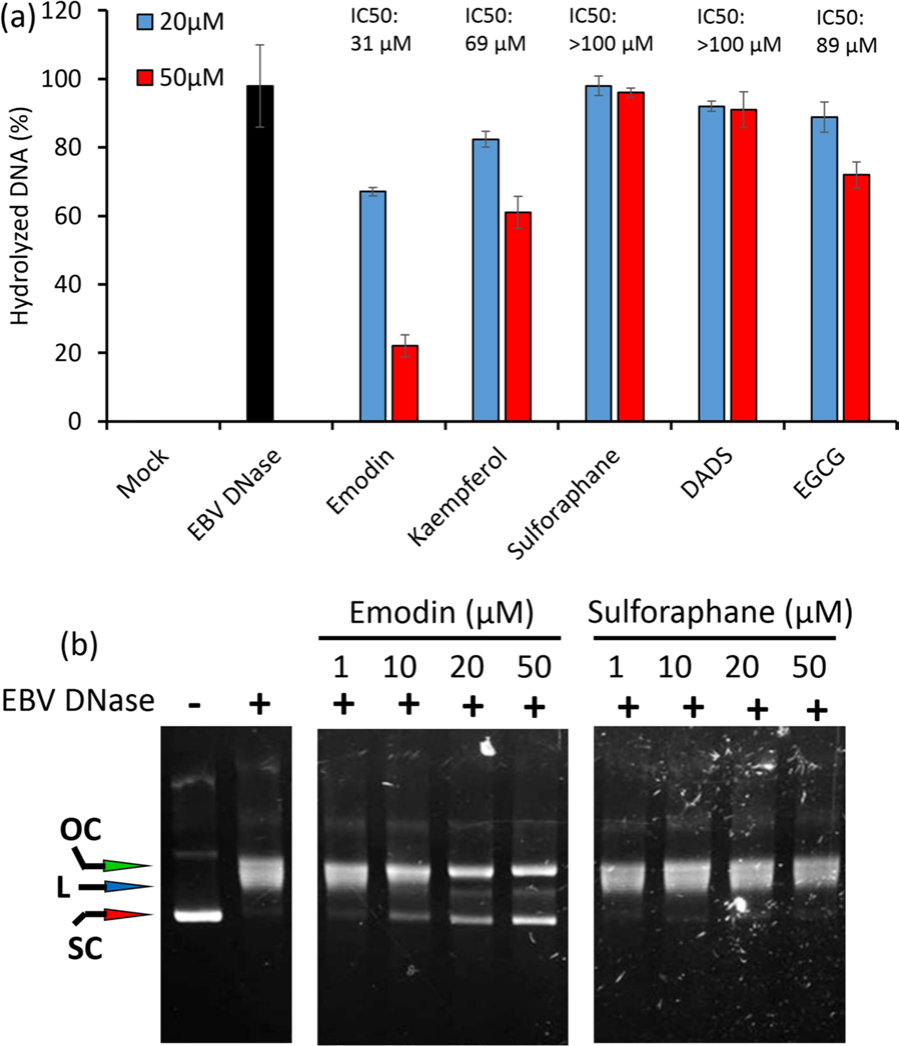
The inhibitory effects of natural compounds on nuclease activity of EBV DNase. **a** Several natural compounds were tested for their inhibitory effect against EBV DNase using the fluorescence-based method. Recombinant EBV DNase was incubated with salmon sperm DNA and the targeted natural compound for 1 h at 37 °Ϲ. PicoGreen dye was then added and fluorescence was detected (excitation: 480 nm, emission: 520 nm). The IC50 values of natural compounds were calculated (top of each panel). The sample treated with the same procedure without enzyme was represented as mock control. Data are presented as the means and standard deviations of three independent experiments. **b** The inhibitory effect of emodin and sulforaphane against EBV DNase was verified using the DNA digestion assay. Recombinant EBV DNase was incubated with pEGFP-C1 and the targeted compound for 1 h at 37 °Ϲ. Samples were analysed by 0.7% agarose electrophoresis. OC, L and SC represent the respective positions of the open circular, linear and supercoiled forms of the pEGFP-C1 plasmid. DADS, diallyl disulfide; EGCG, epigallocatechin gallate

Nuclease inhibitors have potential clinical applications [18], especially in anti-viral therapy [1, 6, 8, 32]. Therefore, we tested several natural compounds using this fluorescent-based platform to identify inhibitors of EBV DNase. As shown in Fig. 3a, emodin significantly inhibited the AN activity of EBV DNase (IC50:31 μM). Kaempferol and epigallocatechin gallate (EGCG) exhibited moderate inhibition (IC50: 69 and 89 μM, respectively), whereas sulforaphane (SFN) and diallyl disulfide (DADS) exhibited poor inhibition of EBV DNase (IC50: > 100 μM) (Fig. 3a). To verify these results, emodin and SFN were further tested using the conventional DNA cleavage assay. As shown in Fig. 3b, EBV DNase was inhibited by emodin, whereas SFN had a lower inhibitory effect on AN activity, which was consistent with the results of the fluorescence-based assay.

Taken together, these results suggest that the fluorescence-based assay can be used for screening AN inhibitors and that emodin is a potential target of EBV DNase inhibitors.

### Emodin inhibits EBV DNase-induced DNA damage and genomic instability

**Fig. 4:**
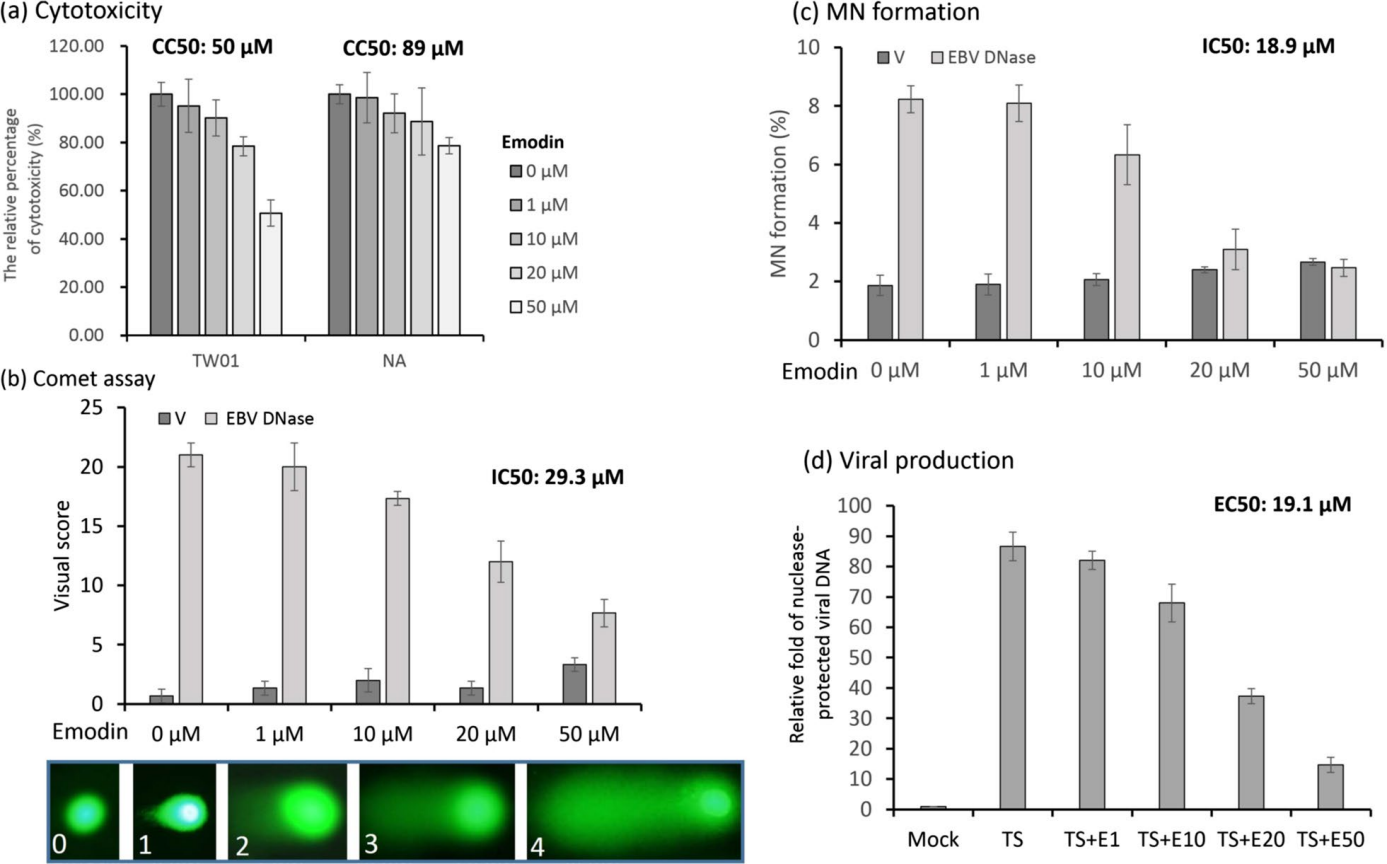
Emodin treatment inhibits DNase-induced genomic instability and EBV production. **a** NPC TW01 cell lines were treated with different concentrations of emodin for 48 h. Then the cytotoxicity of emodin was determined by WST-1 assay. The CC50 values were displayed on the top of each panel. **b** NPC TW01 cell lines were transfected with the vector or EBV DNase-expressing plasmid for 3 h. Then the cells were treated with emodin or mock treatment, respectively. After 24 h of incubation, cells were harvested for the comet assay and MN formation. For the comet assay, harvested cells were mixed with low-melting gel, treated with alkaline solution and separated damaged DNA by electrophoresis. Then the samples were subjected to SYBR Green staining. The comet tail was observed using a fluorescence microscope. Comet assay schemes were quantified using visual scoring. Visual scores are shown as scores of 0–4 indicating an increase in DNA damage (lower panel). The IC50 values were displayed on the top of each panel. **c** For micronuclei (MN) formation, cells were harvested, fixed with methanol and stained with Hoechst dye to count MN. The IC50 values were displayed on the top of each panel. **d** EBV-positive NPC cell lines NA treated with 12-O-tetradecanoyl-phorbol-1,3-acetate and sodium butyrate (TS) with or without emodin. After 48 h, the culture media were collected and treated with DNase I to digest free DNA in the media. The viral genome was released after proteinase K digestion and analysed by qPCR to detect the BALF5 DNA fragment (DNA polymerase). The EC50 values were displayed on the top of each panel. The detailed protocols of all experiments were described in Materials and Methods. Data are presented as the means and standard deviations of three independent experiments. V, vector; EBV DNase, wild-type EBV DNase; TS, 12-O-tetradecanoyl-phorbol-1,3-acetate and sodium butyrate

EBV DNase has important effects on cellular processes and the viral life cycle. It can cause DNA damage and induce genomic instability [56], and plays an important role in viral genome egress and virion production [20]. The comet assay is a standard protocol for detecting DNA damage, and MN formation is an indicator of genomic instability [26]. Next, we performed these two assays to determine the effect of emodin on EBV DNase-induced DNA damage and genomic instability. At first, emodin has been shown a cytotoxic effect on the NPC TW01 and NA cell lines for 48 h treatment. The CC50 values of emodin on TW01 and NA cell lines were 50 and 89 μM, respectively (Fig. 4a). Based on these results, we chose 1 to 50 μM of emodin as the working concentrations for the following experiments. And then NPC TW01 cells were transfected with an EBV DNase-expressing plasmid or vector for 3 h. Then the cells were treated with various concentrations of emodin. After 24 h of incubation, the cells were harvested for comet assay and MN formation. After checking the expression of EBV DNase in each treating group (Additional file 1: Figure S3), the EBV DNase-transfected group without emodin treatment displayed the highest visual score; the comet index revealed robust DNA damage compared to the vector control (Fig. 4b,). However, emodin treatment reduced the visual scores. These results suggest that emodin treatment decreased the comet index in the EBV DNase group, but not in the vector group (Fig. 4b). A similar trend was observed in the MN formation assay. EBV DNase expression induced significant MN formation; however, this phenomenon was repressed by emodin treatment (Fig. 4c). Furthermore, we determined whether emodin treatment inhibited viral production. As shown in Fig. 4d, viral production decreased with increasing emodin concentrations. We further determine whether emodin affects Taq polymerase activity in a PCR reaction. The culture media with 48 h activation were collected and treated as the same as the procedure of viral DNA detection, following with adding for various amounts of emodin to detect EBV BALF5 fragment. The results revealed that the detection values were not affected significantly, suggesting emodin did not inhibit Taq activity in the detection of viral production (data not shown).

These results suggest that emodin can not only inhibit EBV DNase-induced DNA damage and genomic instability but can also inhibit EBV production.

### Docking model of EBV DNase and emodin

**Fig. 5:**
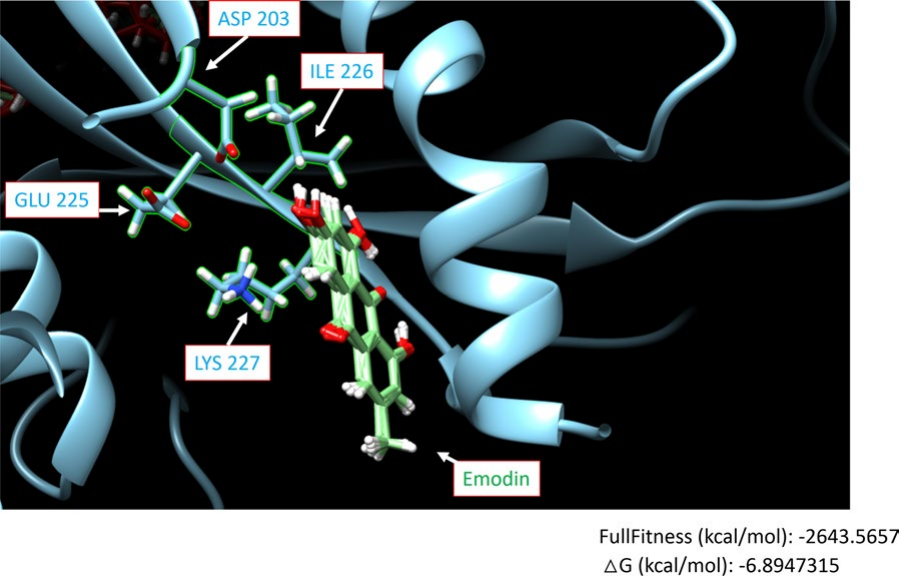
The docking model of EBV DNase and emodin. The possible binding sites of EBV DNase and emodin were predicted using the modelling software SwissDock and UCSF Chimera. The catalytic site of EBV DNase is composed of ASP 203, GLU225, ILE226, and LYS227. The possible docking sites of emodin are shown as green structures inserted into the catalytic cavity

The catalytic site of the EBV DNase was identified as D….EXK motif at positions 203 and 225–227 of the protein sequence, which is responsible for DNA and metal ion binding [38]. We further performed a computational analysis to predict a docking model of EBV DNase with emodin. Because the protein structure of EBV DNase has been resolved [7](PDB ID: 2W45), using Swissdock [23, 24] and UCSF Chimera [41], we predicted a possible docking model in which emodin occupied the catalytic cavity formed by D203, E225, I226, and K227 (Fig. 5). This hypothetical model predicts the possible sites at which emodin interacts with EBV DNase and provides a useful tool to identify other nuclease inhibitors.

## Discussion

The AN of human herpesviruses has been suggested as a potential target for anti-viral therapy [53]. Consistently, small molecules that inhibit the AN activities of CMV [1, 33] and HSV [6, 32] have been identified; however, to the best of our knowledge, no inhibitor of EBV DNase has yet been reported. To explore inhibitors of EBV DNase more easily, a fluorescence-based method for detecting nuclease activity was applied. In the present study, we successfully performed a PicoGreen-labelled method, which has the advantages of convenience, safety, and environmental friendliness for nuclease activity detection, when compared with radioactivity-based assays and DNA cleavage detection by gel electrophoresis (Fig. 1). The optimal conditions for EBV DNase in this assay were determined in terms of pH, temperature, usage of divalent cations, and the influence of ionic strength (Fig. 2). Furthermore, using this platform, we found that emodin has great potential for inhibiting the activity of EBV DNase (Figs. 3 and 4).

Using a DNA cleavage assay analysed by gel electrophoresis, Horst et al. suggested that EBV DNase degraded DNA at pH 7.5 and 37 °Ϲ with 10 mM MgCl_2_ [31]. Using radioactivity-based methods, we previously reported the optimal conditions for EBV DNase activity include 37 °Ϲ and pH 8.0 with 2 ~ 4 mM MgCl_2_ or 200 μM MnCl_2_ [12, 38], while Stolzenberg and Ooka reported 37 °Ϲ and pH 8.0 with 3 mM MgCl_2_ [50], which differ slightly from that observed in the present study (Fig. 2a–2c). Moreover, Stolzenberg and Ooka found that both KCl and NaCl inhibited AN activity at 300 mM [50], compared to 250 mM and 200 mM, respectively, observed in the present study (Fig. 2d). The data variation between the reports of other groups may have resulted from the principal difference in the purification protocol and detection methods. For the application of the PicoGreen-labelled method, because of the similarities of catalytic sites of human herpesviral ANs, this assay can also be used to detect the AN activities of HSV, CMV and KSHV under the catalytic conditions reported in previous studies (data not shown) [21, 29, 47], implying that this method has a broad application. On the other hand, one important application of EBV DNase activity assay is the detection of virus-associated cancers. In the sera of patients with NPC, elevated levels of anti-EBV DNase antibodies can be detected by neutralising DNase protein [10]. Using this fluorescence-based assay, our preliminary results revealed that EBV DNase activity was significantly inhibited by plasma from patients with NPC, but not by plasma of the healthy donors (data not shown), implying that the PicoGreen-labelled nuclease activity assay may be adapted as an EBV DNase neutralisation assay for the diagnosis of NPC.

Recently, the concept of “phytotherapy” has become an attractive approach as anti-viral therapy. The word “phytotherapy” means the application of plant-derived medications in the prevention or treatment of human diseases. In the present study, we focused on phytochemicals and identified potent EBV DNase inhibitors. Emodin was found to exhibit good efficiency in inhibiting EBV DNase activity and EBV DNase-induced cellular genomic instability and EBV viral production (Fig. 4). Emodin and anthraquinone derivatives have been characterized to possess broad anti-viral activity against various viruses, including herpesviruses [1, 2, 17, 54], coronavirus [28, 45], coxsackievirus [39], poliovirus [46] and retrovirus [19, 27, 44]. Emodin has been proposed to use various biological mechanisms to inactivate herpesviruses. For anti-HSV activity, emodin was identified as an HSV AN inhibitor and can inhibit viral plaque formation [32]. Xiong et al. demonstrated that emodin inhibits the replication of HSV-1 and HSV-2 [57]. Emodin and anthraquinone derivatives have been reported to inactivate ganciclovir-sensitive and -resistant CMV [49]. They also dramatically inhibit AN activity and lytic protein expression, suppressing CMV production [1]. Emodin was found to repress the protein expression of EBV lytic genes, thereby inhibiting virion production in B cells and epithelial cells [54, 58]. Emodin exhibits anti-EBV activity by inhibiting Sp1-mediating IE gene expression, and suppressing tumourigenesis in vitro and in vivo [54]. Moreover, in the present study, we demonstrated that emodin could block the activity of EBV DNase, and this may contribute to emodin-mediated inhibition of EBV. Collectively, these studies suggest that emodin is a novel anti-viral agent that exhibits activity against a broad range of viruses and is worth further study to cope with the threat of viruses.

In conclusion, the simple PicoGreen-based assay was suitable to identify natural compounds with the anti-AN activity of EBV DNase. After the screening, emodin was found to inhibit EBV DNase activity and EBV DNase-mediated genomic instability and virion production. We hope that emodin will provide an alternative choice for antiviral therapy through anti-AN activity against EBV DNase.

## Supplementary Information

**Additional file 1. **Supplementary Figure 1. Purification of wild-type EBV DNase protein.  Supplementary Figure 2. Brief overview of the protocols for the radioactivity- and fluorescence-based assays.  Supplementary Figure 3. The protein expression of EBV DNase in emodin-treated NPC cells.

## Data Availability

The datasets used and/or analysed during the current study are available from the corresponding author on reasonable request.
