# Supplementary material for: Screening and identification of emodin as an EBV DNase inhibitor to prevent its biological functions
Source: Virol J. 2023 Jul 13;20:148. doi: 10.1186/s12985-023-02107-x (PMC10339607; doi:10.1186/s12985-023-02107-x)
Supplement: Supplementary file 1 — Additional file 1. Supplementary Figure 1. Purification of wild-type EBV DNase protein. Supplementary Figure 2. Brief overview of the protocols for the radioactivity- and fluorescence-based assays. Supplementary Figure 3. The protein expression of EBV DNase in emodin-treated NPC cells. [file 12985_2023_2107_MOESM1_ESM.docx]

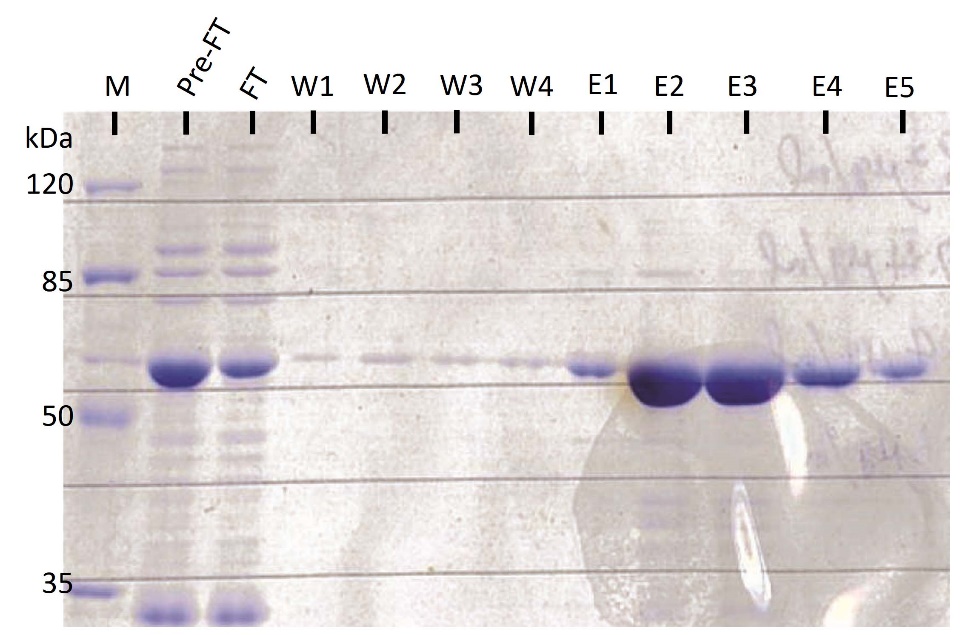


**Supplementary Figure 1. Purification of wild-type EBV DNase protein.**

Recombinant protein of wild-type EBV DNase was purified through a Ni-chelating column and was analysed by 10% SDS-PAGE and coomassie blue staining. The detailed protocol was described in Materials and Methods. The arrow indicates the position of the DNase protein.

M: protein molecular mass markers (kDa).

Pre-FT: pre-flow through lysate.

FT: flow through lysate.

W1 and W2: wash buffer 1 (20 mM Tris/HCl, pH 8.0, 500 mM NaCl, 10% glycerol, 20 mM imidazole).

W3 and W4: wash buffer 2 (20 mM Tris/HCl, pH 8.0, 500 mM NaCl, 10% glycerol, 50 mM imidazole).

E1: elution 1 (20 mM Tris/HCl, pH 8.0, 500 mM NaCl, 10% glycerol, 100 mM imidazole).

E2: elution 2 (20 mM Tris/HCl, pH 8.0, 500 mM NaCl, 10% glycerol, 200 mM imidazole).

E3: elution 3 (20 mM Tris/HCl, pH 8.0, 500 mM NaCl, 10% glycerol, 300 mM imidazole).

E4: elution 4 (20 mM Tris/HCl, pH 8.0, 500 mM NaCl, 10% glycerol, 400 mM imidazole).

E5: elution 5 (20 mM Tris/HCl, pH 8.0, 500 mM NaCl, 10% glycerol, 500 mM imidazole).


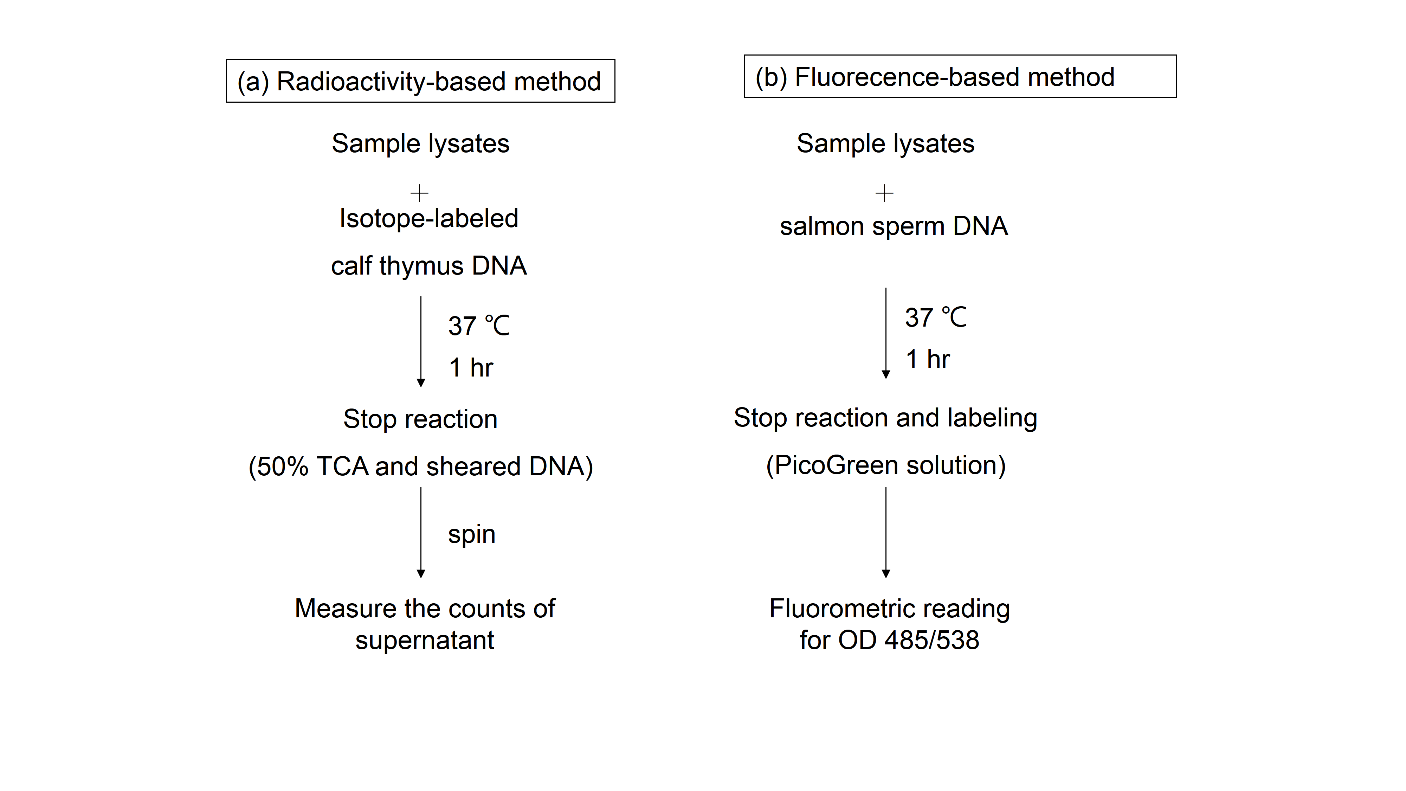


**Supplementary Figure 2. Brief overview of the protocols for the radioactivity- and fluorescence-based assays.**

The major difference between the (a) radioactivity-based and (b) fluorescence-based assays is the use of DNA substrate labelling, isotopes, or PicoGreen. OD, optical density; TCA, trichloroacetic acid.


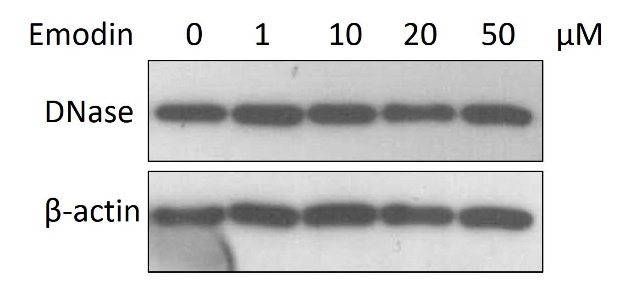


**Supplementary Figure 3. The protein expression of EBV DNase in emodin-treated NPC cells.**

NPC TW01 cell lines were transfected with EBV DNase-expressing plasmids for 3 h. Then the cells were treated with different concentrations of emodin for further　24 h. The cells were harvested for western blot analysis with EBV DNase and β-actin antibodies. The procedure of western analysis were described as Materials and Methods.
